# Ultra-processed food intake is associated with worse mental health in Southern Italian individuals

**DOI:** 10.1093/eurpub/ckac129.553

**Published:** 2022-10-25

**Authors:** G Grosso, J Godos, A Micek

**Affiliations:** 1 Department of Biomedical and Biotechnological Sciences, University of Catania, Catania, Italy; 2 Department of Nursing Management and Epidemiology, Jagiellonian University, Krakow, Poland

## Abstract

**Background:**

A growing body of literature suggests that inclusion of ultra-processed foods (UPFs) in the diet may be associated with various non-communicable diseases, including obesity, cardiometabolic diseases, and increased mortality. However, one of the most underrated research topics is represented by the potential role of diet for mental disorders. The aim of this study was to explore whether there was an association between UPF consumption and mental health outcomes in a cohort of southern Italian individuals.

**Methods:**

Demographic and dietary data from 1572 adults living in southern Italy was collected. Food items were categorized by the level of processing according to the NOVA classification. Multivariate logistic regressions were used to calculate odds ratios (ORs) and 95% confidence intervals (CIs) for the association between UPF intake and mental health outcomes, including sleep quality and depressive symptoms.

**Results:**

Individuals in the highest quintile of UPF intake were more likely to have low sleep quality compared to those with the least intake (OR = 1.54, 95% CI: 1.02-2.34). Among the main component of sleep quality, high UPF intake was associated with sleep latency and efficacy individually. No apparent associations were found between UPF intake and depressive symptoms. However, when considering different age groups, younger individuals (age <40 y) consuming more UPF were more likely to have depressive symptoms than low consumers (OR = 3.50, 95% CI: 1.53-7.99).

**Conclusions:**

These findings show a potential association between UPF consumption and mental health outcomes. Further studies are needed to understand whether the retrieved relation depends on alteration of the physiological feeding patterns leading to food-anticipatory and binge-type behaviors or on nutritional (i.e., high sugars and unhealthy fats) and non-nutritional factors (i.e., additives) triggering pro-inflammatory pathways.

**Key messages:**

• Ultra-processed food consumption is associated with worse sleep quality and depressive symptoms in Italian adults.

• The detrimental associations between ultra-processed food consumption and depressive symptoms seem stronger among younger individuals.

